# A Complete Treatise on the Surgical Anatomy of the Human Body, Both General and Regional

**Published:** 1840-10

**Authors:** 


ART. III.

1. *Traité complet d'Anatomie Chirurgicale, Générale et Topographique, du Corps Humain.* Par ALF. A. L. M. VELPEAU, &c. Troisième Edition, entièrement refondue. *Deux Tomes, avec Atlas.—Paris,* 1837. 8vo, pp. 631, 717.

*A complete Treatise on the Surgical Anatomy of the Human Body, both general and regional.* By ALF. A. L. M. VELPEAU, &c. Third Edition, wholly revised. *With an Atlas.—Paris,* 1837.

2. *Traité d'Anatomie Chirurgicale et de Chirurgie Expérimentale.* Par J. F. MALGAIGNE, &c. Deux tomes.—*Paris,* 1838. 8vo, pp. 480, 664.

*A Treatise on Surgical Anatomy and Experimental Surgery.* By J. F. MALGAIGNE, &c.—*Paris,* 1838.

IT is only within the last few years that surgical anatomy has become a subject of special consideration. The first works which clearly displayed the advantages to the surgeon of a practical acquaintance with the structure of the human body were confined to the description of certain restricted regions, which are especially interested in the performance of particular operations; among these the celebrated treatises of Sir A. Cooper and Scarpa on Hernia, and Langenbeck on the Perineum stand foremost. It is true that some works had been previously published under the designation of surgical anatomy, (for instance that written by John Palfin, which appeared in 1718,) but these were chiefly devoted to descriptive anatomy, and only contained a few detached surgical observations which were unconnected with the anatomical descriptions of the different organs. Sir A. Cooper's and Scarpa's works have been followed by many others, some of which have been confined to the description of individual parts only, while others have embraced the whole frame. In our own language we may particularly mention the work of Burns on the Head and Neck; the first part of a treatise on Surgical Anatomy, by Abraham Colles of Dublin; and Harrison's Monograph on the Arteries. Several manuals of surgical anatomy have been published in Germany, as those of Rosenthal and Bock; but it is in France that this branch of medical literature has been particularly cultivated, most of the complete systems of surgical anatomy being confined to the French language. The works of Blandin and Velpeau have both been long and favorably known. Some of these works have been accompanied by plates; and numerous separate collections of figures have been published illustrative of particular regions of the body, especially of those frequently concerned in surgical operations. Many of these are doubtless very valuable, though we cannot help thinking that the same objection partially applies to them as to anatomical plates generally, that they too frequently supersede the employment of actual dissection instead of merely being used to refresh the memory of the surgeon.

The proper object and limits of surgical anatomy are not very clearly laid down nor perhaps very generally well understood. The greater number of works on this subject have been strictly confined to the relative anatomy of particular organs or parts of the body—or to what is now generally called *regional anatomy*, the object of which is to point out the connexion and relations which subsist between different organs and tissues in any particular region. The principal end of this kind of anatomy is to direct the knife of the operator; and it may be defined as consisting in a correct knowledge of the parts which an instrument will wound or meet with while traversing the body in any given direction. This was originally the sole design of surgical anatomy, but the limits of this study have been much enlarged within the last few years by the French writers, and are greatly extended in the works before us.

The treatise of M. Velpeau has been long before the public, and is very generally known in this country, and we only now place it at the head of the present article on account of the great additions and alterations which have been made in the last edition. When this work first appeared, in 1825, it was solely devoted to the anatomy of regions, but the author has now included many remarks on general anatomy and pathology; his aim being "to explain by the aid of anatomy the numerous points in pathology or surgery which are in any way connected with the appreciable state of organs." The zoologist, the painter or sculptor, the physician, and the physiologist, all require a knowledge of the structure of the human frame, but each needs a knowledge in some respects peculiar to himself. Thus the zoologist looks for the analogies of structure which exist between the organs of man and those of different animals; the painter and sculptor only study the peculiarities of external form; while the physician and physiologist are more particularly interested in the anatomy of the different internal organs or viscera, and in general anatomy or the structure of tissues, by means of a knowledge of which the functions performed by different organs may be elucidated. It is the aim of the surgeon to combine the greater part of this information with an intimate acquaintance with relative anatomy; for "in performing an operation," says M. Velpeau, "it is not sufficient to know the names of the organs which are in danger of being wounded, nor of those which ought to be avoided, the surgeon must also be acquainted with their depth from the surface, their exact connexions, the density and thickness of the layers which connect or separate them, and a thousand other points," which can only be learned by frequent and careful dissection.

M. Malgaigne has extended the scope of surgical anatomy still more widely; for besides general anatomy and pathology, he includes what he calls surgical physiology, and also experimental surgery, which comprehends such points of information as may be obtained by the performance of operations on living animals, as well as from experiments made on the human subject both alive and dead. "Surgery," says M. Malgaigne, "derives its materials from four principal sources: namely, from observation of the living, from the investigation of alterations of structure in the dead, from normal anatomy (acquaintance with healthy structure) applied to pathology, and lastly from experiments." (Preface, p. 1.) These different means of investigation, though concurring to the same end, the greater perfection of the science of surgery, have but little connexion with each other, and have therefore seldom if ever been embraced in the same work. Most treatises on practical surgery include many pathological observations, and numerous surgical remarks will be found in different anatomical works; but few include both anatomy, surgery, and pathology. Experimental surgery has never yet appeared in a distinct form, for though much valuable surgical information has undoubtedly been derived from the performance of experiments on animals and also on man himself, (a fact so well known that it is needless to bring forward any particular illustrations,) this branch of study only exists in the form of scattered facts which are diffused through the various regions of medical science.

The endeavour of M. Malgaigne has been to collect materials from all the above sources, and to mould them into a single treatise, bringing the information derived from anatomy, pathology, experiments, &c., to bear on one common object—the advancement of practical surgery. His success in the accomplishment of this end we rather doubt; for, as might have been expected *a priori*, his work presents a heterogeneous mixture of various matters, the surgical application of many of which is to us difficult of discovery; some appearing solely to relate to general anatomy and others to physiology, &c. It contains, however, much valuable and original information, and an account of many new and interesting experiments. In the present article we shall not attempt to give any analysis of its contents, but shall only endeavour to select those points, both scientific and practical, which appear to possess most novelty, and compare them with the statements of Velpeau and some other writers.

M. Malgaigne enters at considerable length into the minute anatomy of the skin, the structure of which does not seem at present to be completely understood, though many researches have been made concerning it during the last few years.* We shall notice one or two instances in which the structure of the skin bears on surgical pathology and practice.

*Boils.* The seat of the painful affection denominated a boil or furuncle is not universally agreed upon. The dermis or true skin is generally described as being composed of a dense network of whitish fibres, crossing each other in various directions, on the under surface of which a great number of little fossæ or cells are situated between the fibres, each of which cells is filled with a mass of adipose tissue. As we approach the free surface of the skin the network of fibres becomes much more close, so that the external part presents no visible channels of communication with the areolæ or cells beneath, though communications do exist at the points at which the hairs, excretory ducts, &c. escape. These cutaneous areolæ filled with adipose substance, were stated by Dupuytren to be so, and are still commonly supposed to be the seat of boils. In consequence of the suppuration and swelling which occur in these affections the fibres of the skin become separated from one another, and a sieve-like appearance is produced, similar to that which may be obtained by macerating a piece of skin for some time in water. The openings of the excretory ducts, according to M. Malgaigne, become in this state very apparent, and give exit to the matter. In carbuncle, inflammation of a large number of these cells and adipose masses takes place at the same time. The circumscribed form of a boil and the violent nature of the inflammation which always leads to mortification of a portion of the cellular tissue, are partly owing to the fibrous and unyielding nature of the walls of the cells in which the inflammation arises; this produces strangulation and death of the parts concerned.

* See review of the works of Breschet and Gurlt on the Skin, in Brit. and For. Med. Review, Vol. II., p. 429.

Eichhorn* gives a very different description of the structure of the skin. He says that the dermis may be divided into three layers: the middle one of which is hollowed out into a number of little cells containing a watery fluid; he calls these cells lymphatic cavities; they are very minute, he having counted, he says, more than 200 in a square inch. M. Velpeau is of opinion that a boil may arise from the deposition of purulent matter in one or more of these small cells, and he considers that this disease principally differs from carbuncle in the former having its seat in the substance of the chorion, while the latter affects the adipose tissue in the areolæ on the under surface of the skin: this accounts, in his opinion, for the inflammation being much more circumscribed in one affection than the other. The great difficulty, according to M. Velpeau's theory, is to account for the masses of sloughy cellular tissue which are invariably met with in boils, no materials for their formation being found in these lymphatic cavities. M. Velpeau calls them simple lumps (*parcelles*) of concrete pus, but we are at a loss to know how the fluid matter (which he says is first deposited in the cells) can here become inspissated so as to form the tenacious stringy mass which forms the core of a boil.

When inflammation occurs on the inner surface of the skin, M. Velpeau has justly remarked that it has a tendency to spread towards the subcutaneous cellular tissue, where it may give rise to phlegmonous erysipelas. The spreading of the inflammation in this direction is doubtless the principal source of the swelling and tension in boil and carbuncle, a quantity of adhesive matter and serous fluid being deposited in the cellular structure beneath the skin, which thus becomes indurated and produces strangulation of a greater or less portion, which dies from want of nutrition.

The French pathologists make a distinction between the common or benign carbuncle, which they call *anthrax*, and the malignant or pestilential carbuncle, termed *charbon*, which is very similar in its nature to the malignant pustule. Dupuytren regarded the malignant carbuncle as essentially a gangrenous disease; the sloughing of the cellular tissue in this affection arising, according to him, from a peculiar kind of inflammation; while in simple anthrax, on the contrary, he considered it only to be occasioned by strangulation of the processes of cellular tissue extending into the structure of the true skin. M. Velpeau seems to be of opinion that subcutaneous inflammation occurs in malignant carbuncle but not in anthrax or simple boil, in which the inflammation, he says, is confined in the tissue of the skin itself. "Malignant pustule and 'charbon,'" he observes, "originate in the *cellulo-adipose* cavities of the skin, but they almost always extend after a short time, if not simultaneously, to the corresponding part of the subcutaneous tissue; besides which they differ essentially in their cause and nature from furuncle and anthrax." (Vol. i. p. 5.) Our author here confuses furuncle and anthrax together, but in a preceding passage he says "if anthrax differs from furuncle in many respects it is only in consequence of its being seated in the cellular tissue which fills the fossæ on the inner surface of the skin, and not in the substance of the chorion itself." (p. 4.) In both carbuncle and boil the unyielding tissue of the skin causes great irritation and difficulty in the passage of the matter to the surface, and clearly indicates that the most effectual plan of treatment in both of these affections

* Remarques sur l'Anatomie, etc. de la Peau, (Journal des Progrès, tom. vii., p. 88.)

(and one which is imperatively called for in carbuncle where the disease is extensive and produces great constitutional disturbance) is the formation of free incisions through the inflamed part, so as completely to divide the distended skin and to give exit to the matter and sloughs.

*Cutaneous Wounds.* The fibres composing the base of the dermis have been described as interlacing each other in various directions, so as to form an inextricable network; but it seems probable, from the result of some experiments, accidentally suggested in the first instance, that these fibres are arranged in a parallel manner, in a peculiar direction, the course of which varies in different situations. Dupuytren was first led to suspect this fact from observing that several wounds which had been inflicted with a sharp round instrument (*poinçon*) presented the appearance of a longitudinal slit similar to what would be produced by the blade of a penknife. Being struck with this circumstance he made a number of experiments on the dead subject to ascertain if a round instrument puncturing the skin constantly produced elongated wounds: he found that it did, and that the length of the wound varied with the depth to which the instrument was plunged. He also observed that the direction of the wounds was always the same in the same region of the body: thus, on the neck they were directed from above downwards, on the thorax parallel to the course of the ribs, &c.*

M. Malgaigne repeated these experiments with very similar results. He found, however, that the direction of the wounds will sometimes vary a little in the same region in different subjects. Thus, in the body of a female which was covered with a considerable quantity of fat, wounds made on the neck, the anterior part of the thorax, and the abdomen, had always a transverse direction: in a young male subject, on the contrary, wounds on the abdomen followed an oblique direction, corresponding to the fibres of the external oblique muscle; on the chest they ran parallel with the ribs; and on the anterior part of the neck they corresponded with the fibres of the sterno-mastoid muscles. M. Malgaigne says that he has sought in vain for any general law regulating the direction of the fibres of the skin, though he is of opinion that such a law will be discovered by further researches.

"In the skin the direction of the fibres often changes very abruptly. A vertical and transverse wound may be formed by the same instrument within a line of each other. In a few instances I have obtained triangular wounds from punctures with the same circular weapon: this occurred in those regions where the direction of the punctures was observed to change abruptly, and as far as I have been able to determine, this triangular form did not arise from the manner of introducing the instrument, but seemed to be due to the peculiar disposition of the fibres of the skin in particular spots." (Tom. i., p. 67, note.)

M. Malgaigne fancied that perhaps these facts might be applied to some practical uses in surgery: he thought it probable that the retraction of the skin in an operation would be greater from an incision made in a direction transverse to the supposed course of the fibres than in a line parallel with them; but experience has not supported these suppositions; for in a number of incisions which were made with every possible precaution in various situations, the gaping of the wounds appeared much the same in all cases in which the state of tension of the skin was the same.

* Dupuytren, Traité des Blessures, &c. tom. i., p. 61.

Whether the incisions which were made for the purpose of determining this point were practised on the dead or the living subject our author leaves us in ignorance; but if, as we presume, they were made on the former, it must be remembered that the contractile powers of the fibres of the skin would be in a great measure lost after death.

Though from the experiments of Dupuytren and Malgaigne it seems very probable that the fibres of the skin follow particular directions in particular situations, yet this structure at present rests merely on supposition: for neither of these authors nor any other observer that we are aware of, mentions that any peculiar or determinate direction of the fibres of the tissue constituting the base of the dermis, has been ever seen by the microscope.

*Corns.* It is still a point of some doubt whether the cuticle is continued into the sudorific and sebaceous ducts of the skin, as well as the sockets of the hairs. Eichhorn, Lauth, and Gurlt say that it is thus prolonged into the dermis in the form of sheaths; and Malgaigne is of the same opinion. The latter author thinks that these processes of cuticle dipping into the skin form the principal points of attachment between these two tissues. "Is it not," he says, "to a special thickening of these cuticular sheaths that the production of corns is owing—those excrescences of the epidermis which may be distinguished from all others by their possessing a slender root penetrating more or less deeply into the dermis? This is an idea which I have not seen expressed elsewhere, but which appears to me very probable." (Tom. i. p. 72.)

*Topical relation of Nerves to Muscles.* There is a great difference of opinion among anatomists and physiologists as to the place of entrance and the internal arrangement of nervous filaments in muscles. We shall not here enter into the subject of the mode of termination of the muscular nerves, as to whether the fibres anastomose at their extremities or remain distinct. This point, which has been particularly investigated by Prevost and Dumas, Rudolphi, Treviranus, Gottsche, Valentin, Emmert, Dr. Ernst Burdach, and others, has been already treated of at some length in a former Number of this Journal.* Though of considerable physiological interest, it is not of much practical utility. We shall at present confine ourselves to the enquiry respecting the most usual place of entrance, and the primary mode of distribution of nerves in muscles; as a knowledge of these points may be of some surgical importance.

M. Prevost and Dumas describe the trunks of the nerves and their first branches as penetrating between the muscular fasciculi in a tortuous course, the exact direction of which appears to be indifferent. Burdach says "to each individual muscle there goes in general only a single nervous stem. The nerve having entered the muscle, first runs downwards in it to some extent parallel with the muscular fibres."† Neither of these physiologists alludes to the particular part in any of the muscles which the nervous trunks enter. According to M. Lantenois,‡ the nerves which are distributed to the muscles of the limbs commonly penetrate their tissue at their superior or proximal extremities, and quickly divide into two principal branches. M. Velpeau adopts this opinion.

* See Vol. VI., p. 414.

† Beitrag zur Mikroskop. Anat. der Nerven., p. 51; and Brit. and For. Med. Rev. Vol. VI., p. 415.

‡ Thèse Inaug.; Paris, 1826, No. 127.

"Each muscle," he says, "commonly receives one nervous cord of a certain size, which enters a little above the middle third, and immediately divides into two branches, one of which supplies the superior and the other the inferior part of the organ." (Vol. i., p. 61.) M. Chassaignac,* who paid particular attention to the nerves of the superior extremities, informs us —1, that each large muscle receives many nervous filaments, sometimes from the same trunk, and at other times from different branches; 2, that those muscles which are composed of several fasciculi of fibres, are supplied with distinct branches for each of such fasciculi, and if the different fleshy portions of the muscle are of unequal lengths, each of them receives nervous filaments which come off from the principal nervous trunk, at different corresponding heights; and 3, that every muscle terminating in several tendons but having a single fleshy belly receives several nervous filaments which generally correspond in number to its tendons.

With respect to the mode of entrance of the nervous filaments, M. Chassaignac says that every muscle receives its nerves by that surface which is next to the axis of the limb, and that most muscles are penetrated by their filaments in their superior fourth. M. Malgaigne has carefully investigated this subject for the purpose of determining whether the statements of M. Chassaignac are correct, and he has arrived at different conclusions with respect to the general place of entrance of the nerves of the limbs. He says that the nerves of the arm always penetrate the muscles below their superior fourth, and in many cases as low down as the base of the superior third or even lower. In the thigh, however, he has found some of the muscles penetrated by their nerves within their superior fourth; thus in a sartorius measuring 21 inches in length, taking the fleshy and tendinous portions together, the nerve entered at the distance of four inches from its proximal attachment or origin. M. Malgaigne found that it was impossible to establish any general rules by which the precise point of insertion of the nerves in muscles may be known, for this seems (as might be supposed from the discrepancy of the foregoing statements) to vary considerably in different individuals. The only conclusions which he arrived at were—that nerves scarcely ever penetrate the muscles of the limbs in either their superior or inferior fourth: the knowledge of this fact, if fully established, may be of some surgical value; though we fear, notwithstanding the contrary opinion of M. Malgaigne, that the point of entrance is too variable ever to become of practical importance to the surgeon.

"Suppose, for example," says our author, "that a sabre-cut on the inner side of the thigh transversely divides the *gracilis* muscle three inches from its pubic end, the upper part will here be paralyzed, and convulsions, if they should come on, will only affect its inferior part. If the division, however, take place an inch lower, the phenomena will be exactly reversed. If the nervous trunk supplying the muscle shall have been itself divided the muscle will be entirely paralyzed." (Vol. i., p. 101.)

The division of the nerves supplying a muscle was ascertained by Bichat to have no influence over its contractile power; but Velpeau was not aware of this fact, for he states that the muscles of the thigh undergo a much greater degree of retraction when amputation is performed below the point of insertion of their nerves, than when above it. Malgaigne

* Compte rendu des Travaux de la Société Anat. 1832.

explains the fact of the retraction of the muscles being greater when divided at a distance from their origin by the greater length of their fibres.

*Relation of Blood-vessels and Muscles.* Muscles are abundantly supplied with both arteries and veins. It has been observed that the latter are furnished with very few valves, and that they may be filled with injection, impelled from the nervous trunks towards the small branches. It is generally considered that the necessity for the presence of valves is obviated by the assistance which the muscular contractions afford to the propulsion of the blood through the veins traversing the substance of the muscles. Bichat founded this opinion principally on the well-known fact that the stream of blood escapes more quickly from the arm of a patient during venesection when he contracts the muscles of the limb than when they remain in a state of relaxation. M. Malgaigne says that this circumstance must be referred to quite a different cause.

"The contractions of muscles," he remarks, "render their tissue more close, and prevent the veins from admitting their ordinary quantity of blood; it is in consequence of this that it flows into the superficial veins. During any violent effort all the veins on the surface of the body become swelled, the capillaries even carry red blood, whence it is evident that the circulation is impeded. This excessive repletion frequently renewed is certainly one powerful cause of dilatation and irritation in the superficial veins, especially in those in which the blood likewise stagnates by its own weight; thus *varices* are much more common in men than women, and in the legs than anywhere else. The muscular veins which are emptied by the least effort are on the contrary very rarely affected with varices." (Tom. i., p. 99.)

We are inclined to think that this explanation is correct; for though the venous circulation is undoubtedly aided by the action of the muscles in those vessels which are furnished with numerous valves, yet this cause cannot operate on the muscular veins, which we have already stated to be nearly destitute of valves, and in which therefore intermitting pressure will force the current of blood backwards as well as forwards. M. Velpeau, when speaking of the contrivances by which the large arterial trunks are prevented from being compressed by the contractions of muscles, remarks that "it is less by pressure on the trunk than by momentary obliteration of the arterial branches which are distributed through their substance, that the muscles force the blood towards the internal cavities or the surface of the body, when they contract with a certain degree of force." (Tom. i., p. 60.) This observation of M. Velpeau, as to the compression of the arterial branches by muscular contraction, coincides in a great measure with some of the doctrines of Mr. Wardrop* respecting the influence exerted by the muscles over the circulation, and which he brings forward as something new. Mr. Wardrop, however, considers that though the flow of blood is impeded through the arteries by muscular contraction, it is propelled onwards, on the contrary, in the veins. He makes no distinction, however, between the superficial and deep-seated, though as the latter are destitute of valves they will be quite as much under the influence of pressure as the arteries. We agree with M. Malgaigne that the effect of muscular contraction is to force the blood into the cutaneous veins, and in this way we must account for the more ready flow of

* On Diseases of the Heart. See Brit. and For. Med. Rev., Vol. V., p. 146.

blood from the arm when a solid body is firmly grasped in the hand during venesection.

*Rupture of Muscles and Tendons.* Living muscular fibres have generally been considered to possess a greater degree of strength and resistance than tendinous fibres; experiments and facts, however, show that this is only partially true. M. Malgaigne thinks that the tenacity depends upon the fibres being in a state of contraction: for when a violent degree of extension is applied to a limb, the muscles of which are in a state of relaxation or stretched and elongated, as in reducing a dislocation by means of pullies, or when a limb is torn off by machinery—he says it will be found that the muscles generally give way through their fleshy portions, and that their tendons will bear a much greater degree of force without breaking. On the contrary, any sudden force applied while a muscle is in a state of powerful contraction will snap the tendons and not the fleshy fibres. It is in this way that rupture of the tendo-achillis takes place.

This explanation is only partly applicable, for it has been found that the relative strength between different muscles and their tendons varies greatly, so that in some muscles the fleshy fibres will rupture, while in others the tendons will break upon the application of the same degree and kind of force.

Ruptured or divided tendons are generally a long while in uniting, the slowness of cicatrization being principally owing to the difficulty which is always met with in keeping the separated portions in contact. Velpeau says that when the contact is perfect, union mostly takes place very readily.* Tendons possess but a feeble degree of vitality, being usually described by anatomists as destitute of visible vessels and nerves.† In consequence of this low state of organization, they may be cut or wounded without pain and without much risk of producing inflammation, so that Velpeau remarks that a suture may be passed through them with perfect security.

"The suture," he says, "the only means of keeping the ends in contact, which modern surgeons have banished from practice, does not deserve the contempt into which it has fallen. The pretended dangers which it was supposed liable to give rise to in such cases are purely imaginary: traversing a solid tissue possessing but little irritability it unites the divided parts with as much force as is required." (Vol. i., p. 73.)

M. Velpeau does not say whether he has himself put this in practice; there is no doubt that it might be advantageously resorted to in cases where there is a difficulty in keeping the divided ends of a tendon in contact, and when it is desirable that this should be done: an event which can rarely occur, as laying bare a tendon for the sole purpose of applying a ligature seems scarcely in any case desirable. The only cases in which it might be supposed to be beneficial are those in which tendons have been completely divided by a cutting instrument, and where the retraction of the muscles prevents the separated extremities from being brought into contact. This sometimes occurs in cases in which the

* M. Stromeyer has found union to have commenced as early as the fifth day, in cases where the tendons have been surrounded by a sheath, which has kept the divided ends in contact.

† Mr. Paget has lately succeeded in injecting the vessels of tendons. See Medical Gazette, July, 1839; and Brit. and For. Med. Rev., No. XVI., p. 594.

extensor tendons of the fingers have been divided on the back of the hand.

We have stated that when the ends of a divided tendon are separated by a considerable interval, the process of union is some time before it is completed, for a portion of new substance has to be generated. The changes by which cicatrization is effected in these cases have been minutely studied by Von Ammon, Bouvier, and others, and have been before briefly alluded to by us in former Numbers.* As, however, they are of considerable practical importance, we shall once more describe them, in the words of M. Malgaigne, to whom M. Bouvier communicated the results of his experiments before he published them, and who carefully examined his preparations.

"The tendo-achillis having been divided in several dogs: on the second or third day after the cellular sheath of the tendon was found thickened and formed a kind of canal, which was complete except at the place by which the knife had entered. The inner surface of this canal was of a bright red colour, as well as the divided ends of the tendon. In some cases the canal was empty, in others filled with coagulated blood. By the ninth day the sheath had become very much thickened, and already formed a very firm uniting medium between the separated ends of the tendon; the canal was now much contracted, and the aperture by which the knife had entered was obliterated. From the twelfth to the eighteenth day the new substance presented the appearance of a cord of the same size as the tendon itself, and which had begun to take on a fibrous structure. By the twenty-fourth day the canal had entirely disappeared; the intervening cord was manifestly fibrous, but of a grayish colour, and very different in appearance from the tendinous tissue to which it solidly adhered. In many cases the new substance was more gray towards the centre and was considerably swelled at its extremities. M. Bouvier thought at first that this swelling was owing to some expansion of the tendinous tissue itself, but on making a longitudinal section of one of his preparations we found that the tendon was scarcely increased in thickness, almost the whole of the tumour being formed by a circular thickening of the sheath, which enveloped each extremity of the tendon, in the same manner as the provisional callus envelopes the broken ends of a bone in a fracture. These swellings were not always met with, and they seem to disappear in the process of time; so that the phenomena accompanying this union of tendons are very analogous to those attending the formation of osseous callus, and they may be referred to the same laws." (Tom. i., p. 126.)

These experiments are very interesting; but M. Malgaigne has not described the appearances presented by the new or intermediate substance beyond the 24th day. At a later period does the grayish colour disappear, and does the connecting medium present the characteristics of genuine tendinous structures? M. Bouvier says not: the cicatrix of the tendon of a horse after two months was still of a grayish colour; that of a dog after an interval of six months, he says, presented a very distinct and separate structure, but he does not mention whether it retained any vestiges of the gray colour.† Müller says "the substance which reunites divided tendons has not, it seems, the fibrous shining aspect of tendons but is more cartilaginous." From these observations we conclude that the gray colour which probably depends on the degree of vascularity of the new tissue ultimately disappears, though the structure of the tissue remains different.

* Brit. and For. Med. Rev., Vol. V., p. 550; Vol. VII., p. 240.

† Bull. de l'Académie de Médecine, tom. ii., p. 703.

*Fracture.* When treating of the interesting subject of the mode of union of bones, which has excited so much attention among physiologists and surgeons, M. Malgaigne remarks that the theory of Dupuytren, so generally admitted, respecting the formation of a provisional callus, before the true uniting medium or permanent callus is deposited between the ends of the bones, is only admissible in a certain number of cases, as in the hollow cylindrical bones of adult animals. M. Malgaigne says that in young subjects the union of fractured bones takes place immediately, and without the formation of any provisional callus; and that in fractures of epiphyses and short bones, in which, from the want of a medullary cavity, there can be no deposition of callus in that situation, it is also frequently absent externally, and may always be prevented from forming by external pressure. M. Velpeau seems to entertain nearly the same views, considering that union takes place in different bones in different ways, on account of the difference of arrangement which exists between the vessels by which they are supplied with blood. One order of vessels traverses the periosteum and penetrates into the interior of the osseous tissue; another set forms a fine network between the periosteum and the bone, and principally nourishes the external osseous layers.

"The provisional callus described by Dupuytren and others," he says, "appears to be entirely dependent on these latter vessels, while the definitive callus, the cellular cicatrization of the fractured extremities, must be referred principally to the former set. It is this circumstance which renders the work of consolidation far from the same in all bones. When the vascular network of the periosteum predominates, as in the shafts of the long bones which are furnished with a large medullary canal, the provisional callus generally forms in the manner described by authors. In the spongy bones where the first-mentioned vessels predominate, the ideas of Bardenave and Bichat* are applicable rather than those of Dupuytren. Lastly, if (as in the instances published by M. Gaillard† and as I have myself observed a great number of times in the fibula and tibia, and the radius and ulna,) the ferule admitted by the disciples of Dupuytren is absent when the coaptation and immobility of the fragments are exactly maintained, it follows that when the work of reparation which is undertaken by the vessels belonging to the osseous tissue itself is uninterrupted, the superficial vessels are not required." (Vol. i. p. 148.)

According to these views of M. Velpeau, which coincide with those of Mr. Syme, deduced from his own experiments, (see our Eighth Vol., p. 285,) the periosteum and its vessels must play the sole part in the formation of the provisional callus: it is, however, now most generally admitted that all the vessels of the part, those of the bone, periosteum, and adjacent structures, seem to be equally concerned in the process. It is remarkable, considering the number of experiments which have been made on the mode of union of bones by so many and such able men, that there should still be so much obscurity remaining. The greater number of observations have been made, however, on adult animals, and on the cylindrical bones; and it is by no means improbable that the short spongy bones may unite without the formation of any provisional callus: the same may also be the case in young animals whose bones possess a much greater degree of vascularity; but we cannot agree with M. Velpeau

* These anatomists make the union of fractures analogous to that of the soft parts by means of granulation.

† Revue Méd., 1830, tom. i., p. 67.

in thinking that union takes place in these cases by means of granulation. Why is it not effected by means of coagulating lymph, which uniting the broken fragments and becoming organized may be finally converted into bone? There is room for the institution of fresh experiments on this subject; and they should be particularly directed to the observation of the mode of union of the short and flat bones, as very few researches have been made respecting these parts of the osseous system. In several experiments which we have ourselves instituted on rabbits, and in which the bone selected has been the scapula, we have found union to be effected by means of provisional callus deposited in considerable quantity along the line of fraction, and reparation has been effected in this manner in cases where there has been no displacement of the fractured portions of bone.*

*Structure of Arteries.* The arteries are generally described as consisting of three coats: an external, middle, and internal. M. Malgaigne says, however, that this statement is only correct when applied to the pulmonary arteries, as in those belonging to the aortic system four may be distinguished. The external coats of all the arteries is formed of condensed cellular tissue, the fibres of which are closely interwoven, and are generally described as becoming more firmly connected to each other (rendering the tunic more dense) as it approaches the middle coat, to which it is closely connected. Lasône,† with whom M. Malgaigne seems to agree, says that this structure is only met with in the adult male subject, for that in women and children the structure of the cellular coat is equally loose throughout. Some anatomists, particularly the older ones, describe an intermediate tunic between the external and middle ones. which they denominate nervous or tendinous: Lasône remarks that this exists in the ox though not in the human subject, and thus the older anatomists were perhaps led into error by deriving their knowledge of the human body from the dissection of animals.‡

The second or middle tunic consists of fibres running in a transverse direction, which are described as not of sufficient length to form perfect rings round the whole circumference of the vessel, but as being short and rather oblique in their direction, and interlacing at their extremities between the neighbouring fibres.§ M. Malgaigne differs from this view, and describes the fibres as running in a spiral direction, each fibre coiling several times round the artery. He says that having made a small perpendicular incision through the middle coat of the aorta (the cellular tunic having been first carefully removed), he unrolled with great care those fibres which had been divided by the knife, and thus he succeeded in obtaining some of them several inches in length, which made two or three turns round the artery, mounting in an ascending spiral direction. This statement may be perfectly correct, and yet not interfere with the

* Mr. B. Cooper has been lately occupied with the subject of the reparation of bone, and he has given the results of some of his experiments in the Fourth Number of the Guy's Hospital Reports, which have been noticed by us in our Fourth Volume. His experiments, like those of his predecessors, have been confined to the long bones.

† "Sur la Structure des Artères," Mém. de l'Acad. des Sciences, 1756.

‡ We can find no mention of this peculiarity of structure in the works of Cuvier, Meckel, or other comparative anatomists.

§ Cyclopæd. of Anatomy, vol. i., p. 222.

truth of the previous description; for in all probability those portions of the tunic which M. Malgaigne unrolled were not single fibres but bundles of fibres, consisting of short oblique filaments interlaced together. Our author does not mention having examined them with the microscope. The spiral disposition of the tissue composing the middle coat of arteries has been previously described; Beclard alludes to it, though he says that he has never himself observed it.

Malgaigne adheres to the opinion of Haller and Hunter, that the middle coat of arteries is muscular. He admits that a vast difference undoubtedly exists between its texture and that of the sartorius or any of the larger muscles, but that scarcely any distinction will be observed between its fibres and those of the muscles of the external ear. A still closer resemblance, it has been said, may be observed between the fibrous arterial coat and some of the involuntary muscles, the muscular coat of the intestines for instance.* M. Malgaigne supports his views on this subject with considerable ingenuity; but, as we think that there are few anatomists who will agree with him at the present day, we shall not enter into his arguments. M. Malgaigne describes a distinct tunic as existing between the fibrous and inner coat of arteries: he denominates it *sub-serous.* Haller mentioned it under the name of *tunica cellulosa interior*, and by most modern anatomists it is considered as forming only the outer layer of the inner coat. M. Malgaigne says that it only exists in the aortic system of arteries, the pulmonary artery and its branches being unprovided with it. He says that its appearance varies according to the size of the arteries in which it is examined. In the aorta it is white, thick, opaque, and fragile, does not present any appearance of fibres, but may be detached in layers, several of which seem to exist. In the smaller arteries it becomes soft and cellular, losing its whiteness and brittleness, and is thrown into wrinkles by the contractions of the fibrous coat. It is more difficult to distinguish this sub-serous coat in the arteries of women and children than in men. Our author considers that the large arteries owe the power of preserving their cylindrical form, and of presenting an open mouth when cut across, in a great measure, to the solidity of this tunic. He says that it does not exist in the pulmonary artery, and it is therefore that this collapses much more on division than the aorta and its large branches. It is in this tunic that the steatomatous, cartilaginous, and osseous patches so often met with in the aorta and its branches are generally considered to be deposited: Haller remarked this circumstance. By those authors who do not admit of the existence of this fourth tunic these morbid deposits are described as being seated in the outer layers of the inner coat, or between that and the middle coat. M. Malgaigne says that it is remarkable that the pulmonary artery appears never to be the seat of these deposits: Bichat was struck with this circumstance, and endeavoured to attribute it to some difference in the nature of the lining membrane; but Malgaigne considers that the real cause will be found in the difference of anatomical structure which we have mentioned, namely, the absence of the sub-serous coat. According to a recent observer,† the cartilaginous patches met with on the inner surface of arteries must

* Skey, Philos. Trans., 1837.

† M. Bizot in Mém. de la Soc. méd. d'Obser.; and in Brit. and For. Med. Review, Vol. VI., p. 46.

not be confounded in situation with the steatomatous or osseous deposits which also occur; for he says that the former are not contained at all beneath the lining membrane of the arteries, but are developed on the free surface and in the tissue of the inner coat itself. Mr. Mayo also makes the same statement.* If the view of these latter pathologists is correct we must look for some other peculiarity in the structure of the pulmonary artery, besides the absence of the sub-serous coat, which exempts it from this morbid alteration. M. Louis states that this exemption of the pulmonary artery from these morbid deposits must be attributed to the properties of the blood circulating through it; for in some cases, where, from congenital malformation, there has been a mixture of the blood of the two sides of the heart, this vessel has been found diseased.

The inner coat of arteries is smooth and polished, and in appearance very closely resembles the serous membranes, particularly the arachnoid membrane of the brain: when examined with the microscope it has been said that its inner surface may be seen to be furnished with a number of villi, giving it a velvet-like appearance. This, according to Cruveilhier,† is owing to a mucous layer which is spread over the interior of the vessel in the form of an extremely fine pellicle. This surface secretes an unctuous fluid, which lubricates the interior of the vessel and enables the blood to pass smoothly through it without adhering to its sides. M. Velpeau says that both the villous surface and its secretion inevitably disappear as soon as this tunic becomes the seat of the least morbid alteration; so that if these characters are met with after death we may affirm that the arteries have not been inflamed. If this statement may be depended upon we shall have a certain test by which inflammatory congestion of the arteries may be distinguished from simple staining of the lining membrane by the imbibition of blood after death. M. Velpeau says that the lining membrane of the arteries possesses all the attributes of an inorganic layer.

"It is a sort of varnish of horny or epidermic matter, which exhibits no traces of vessels or nerves. It is thus altogether insusceptible of primary inflammation, and all that has been said of diffused arteritis, either acute or chronic, may be referred to pure supposition. The more or less deep red tints which are so often observed, are the phenomena of imbibition, which depends on the qualities of the blood, the kind of death, and the state of the atmosphere." (Vol. i., p. 80.)

M. Velpeau thus agrees with Bizot,‡ Trousseau,§ Louis, and Andral as to the pathological value of red coloration of the internal surface of the arteries. With respect to his views as to the inorganic nature of the lining membrane, we confess that we are rather sceptical. The observations of Eulenberg|| have shown that there exists a fine layer of epithelium composed of roundish or rhomboidal scales, which lines the whole common internal membrane of the blood-vessels. The villi described by Cruveilhier have not been observed by other microscopic anatomists.

*Compression of the Brain.* Taking leave of general anatomy, we shall next draw the reader's attention to a subject which must be placed under the head of experimental surgery; and on which M. Malgaigne has been making some interesting researches—namely, the effect which is produced on the brain by compression, particularly that compression which is

* Outlines of Pathology, p. 448. † Dict. de Méd et de Chir. Pratiq. Art. *Artère.*

‡ Ibid. § Arch. gén. de Méd. t. 14. || Henle ueber die Ausbreitung des Epithelium.

caused by the presence of effused blood, or other fluid within the cranium, lessening its cavity.

M. Malgaigne is of opinion that in most cases a much greater degree of importance is attached to this lesion than it deserves; and he thinks that the bad symptoms which are so frequently referred to compression simply are rather owing to some injury of the brain itself, such as concussion, occasioned by the same accident as that which has given rise to the effusion of blood. It is scarcely possible to conceive any rupture of the vessels of the dura mater or pia mater taking place from a blow (not fracturing the skull), without the brain being at the same time seriously shaken. For the purpose of ascertaining directly the effect of pressure on the brain, numerous experiments have been made on living animals.

M. Serres trepanned a dog over the track of the superior longitudinal sinus, and after making an opening with the bistoury through the sinus, closed the external aperture in the skull, so that none of the effused blood should escape externally. The animal being let loose after this proceeding, ran about as if nothing had happened, and continued without any bad symptoms for three hours, at the expiration of which time it was killed, when a very large clot of blood was found in the great fissure between the two hemispheres, and another of less extent on the surface of the left hemisphere of the brain. This experiment was repeated by M. Serres upon other dogs of different ages, upon rabbits, pigeons, &c., and always with the same results.*

It has been objected to these experiments that the degree of compression could not be accurately known, in consequence of the pressure of the cranium being partially removed by the operation of trepanning. M. Flourens therefore repeated them on young pigeons, in whom the skull was so soft that he was enabled to penetrate the sinuses of the brain with a sharp instrument without removing any portion of the bone. He found that under these circumstances the effusion of blood produced loss of sight, convulsions, and sometimes death. He also found that the removal of a portion of the skull instantly relieved these symptoms.†

Neither of these sets of experiments possesses much value, because the relative quantity of effused fluid, in regard to the capacity of the cranium, was not at all ascertained, and this, as we shall presently show, is a point of great importance, the dangerous effects of pressure appearing to depend entirely upon the degree to which it is exerted. M. Malgaigne relates an experiment to exemplify this point, which he refers to Sir A. Cooper, but we have not been able to find it recorded in the writings of that celebrated surgeon. The experiment was as follows: the skull of a dog having been trepanned, direct pressure was made on the brain through the opening with the finger. At first the animal seemed scarcely to feel it, but on increasing the pressure the dog showed symptoms of pain and irritation, and endeavoured to escape. The pressure being gradually increased more and more, the dog became comatose, and fell down; the pressure was continued for five or six minutes, and then the finger was removed, upon which the animal revived, and soon walked about as well as before the experiment.

* Annuaire Medico-Chirur. des Hôpitaux, 1819, p. 246.

† Archives gén. de Médecine, tom. xxv., p. 133.

For the purpose of ascertaining what quantity of fluid might be effused within the cranium without producing serious symptoms, M. Malgaigne instituted some fresh experiments. He first injected half an ounce of warm water into the cranium of a small dog (it is not stated whether the fluid was effused between the cranium and dura mater, or between that membrane and the brain). The animal was immediately seized with convulsions, followed by profound coma, which continued till a free opening was made into the skull. The quantity of fluid injected in this instance seemed but small, but it was very considerable when compared with the size of the cranium, the cavity of which when measured was only capable of containing two ounces of water. M. Malgaigne next took a rabbit, and having made an aperture in the skull with a bodkin, sufficiently large to admit the tube of an Anel's syringe, he injected about a drachm of tepid water, which instantly produced convulsions and death. It was afterwards ascertained that the cranium would only hold two drachms and a half of fluid. In the third experiment he took a rabbit whose cranium would contain 170 grains of water, and he injected into it, between the dura mater and the brain, fifteen grains of fluid; this produced slight anxiety of respiration, which, however, only remained for a few seconds. At the end of six minutes he injected thirty grains more, which produced no other effect than slight diminution of vivacity. Another thirty grains being injected after an interval of a few minutes, the animal appeared stunned, and the respiration became laborious; it completely recovered, however, in less than half a minute. A fourth injection of another thirty grains was made after a short interval; and M. Malgaigne was proceeding to inject more, when a convulsion came on, and the rabbit fell down in a state of insensibility. At the end of half an hour it slightly recovered, when a fifth injection of thirty grains of liquid was made, which brought on a violent convulsion; the animal sprung up more than a foot into the air, and on falling down continued to be affected with slight convulsions. M. Malgaigne says that he now injected another forty-five grains of water, upon which the respiration immediately ceased, and the eyes seemed starting out of the head; the heart, however, continued to beat for a long time. On examination of the body after death, only a few drops of fluid were found in the skull, but a considerable quantity escaped from the spinal canal; this was not measured, though M. Malgaigne thinks that it was much below the quantity injected. In this experiment great care was taken that none of the fluid should escape from the orifice by which it was introduced, and it actually required the injection of a greater quantity of liquid than the cranium itself was capable of containing to kill the animal. It must be taken into account, however, that it was introduced gradually, and at several distinct periods, and also that it was injected between the brain and dura mater, so that the fluid could penetrate into the spinal canal; M. Malgaigne concludes that great part of the fluid must have been absorbed during the performance of the experiment, which altogether occupied an hour.

M. Malgaigne deduces from these and other experiments that compression of the brain, without any lesion or injury to its substance, is only dangerous when it exceeds certain limits; and states it as his conviction that "the doctrine held by the 'Academy of Surgery' upon the necessity of trephining, in cases of compression of the brain after wounds of the head (without depression of bone), is an old and deplorable error, which even causes numerous victims at the present day." (T. i., p. 317.)

The capacity of the cranium of an adult man has been estimated at about forty-four ounces, and of a woman at from thirty-eight to forty ounces. Sir A. Cooper says that the greatest quantity of blood that he has ever found effused after death was about three ounces; and such a quantity as this is not at all likely to be extravasated within the skull from a blow without rupture of some considerable artery, as the middle meningeal, which, Malgaigne says, can scarcely take place without fracture. "Such an effusion as this will only reduce the brain of a man one fifteenth in volume, and it has been supposed that such a slight compression may give rise to coma, paralysis, and death!"

This opinion that pressure on the brain, unless carried to a very considerable degree, does not produce any important consequences when uncomplicated by other injuries, is supported by facts recorded and observations made by many authors. Mr. Abernethy thought that the recovery of many cases which he had met with of fracture of the cranium (in which there was depression of a portion of bone) without the performance of any operation, proves that at all events a slight degree of pressure may not derange the functions of the brain for a limited time after its application, and probably never; for all those patients whom he had an opportunity of knowing for any length of time after such an accident as the above, continued as well as if nothing of the kind had happened to them.* Sir B. Brodie says "in whatever way compression of the brain operates, so as to disturb the functions of that organ, it is difficult to explain wherefore the symptoms to which it gives rise are sometimes slight, and at other times urgent: symptoms, not to be accounted for by any differences in the quantity of pressure, nor in the particular part of the brain affected by it. At the same time it is undoubtedly true that for the most part the patient suffers more from an extensive than from a slight depression, more from a large than a small extravasation."†

M. Malgaigne would account for the differences between the symptoms in these cases by the presence of some lesion of the brain itself in some of the patients and not in others. At any rate the facts adduced (and they might be almost indefinitely increased) ought to make surgeons very cautious in the use of the trephine; the application of which instrument might, with advantage, be almost exclusively limited to those cases in which there is evident depression of bone, accompanied with symptoms of compression.

*Affections of the Vertebral Column.* The degree of mobility of the vertebral column chiefly depends on the structure and disposition of the intervertebral fibro-cartilages. The thicker these are in proportion to the thickness of the bodies of the vertebræ, and the less the size of the articulating surfaces, the more mobile will any part of the spine be. The intervertebral substances are said by M. Malgaigne not to be at all liable to ossification, but according to him they are frequently affected with

* Abernethy's Surgical Works, vol. ii., p. 4, &c.

† Med.-Chir. Trans., vol. xiv., p. 344.

other morbid alterations, which have not, he thinks, been sufficiently attended to.

"Sometimes these alterations do not occasion any change in the direction of the vertebral column, because nature causes a growth from the edges of the bodies of the neighbouring vertebræ, which thus become connected together by their circumference, and prevent any sinking of the fibro-cartilages, and consequent deviation from the natural position of the spine. In most cases, however, sinking takes place; the bodies of the two neighbouring vertebræ approach each other in front, while their spinous processes separate behind and form an unnatural projection. This is by no means a rare variety of Pott's disease." (Tom. ii., p. 17.)

According to our author, those lateral curvatures of the spine, commonly referred to rickets, are principally owing to some morbid alteration of the intervertebral layers, in consequence of which the vertebræ deviate from their straight position, the bodies approximating more closely on one side than on the other. M. Malgaigne thinks that this affection depends on a general want of nutrition of that side of the body on which the sinking takes place, and supposes that this inequality of nutrition may be either original and congenital or acquired. In support of this theory he refers to the researches of M. Maisonabe, who has observed that more than half of the subjects affected with lateral curvature of the spine have been affected during the first years of life with convulsive movements and great nervous excitability; and that two thirds of them have one eye smaller than the other, or one side of the face; and in many instances the whole of one side of the body less perfectly developed than the opposite.* If there is any defect of nutrition in these cases, the vertebræ themselves should participate in the disease; and Malgaigne allows that they do so in some degree, though to a much less extent, than the fibro-cartilages; the latter giving way more readily under the pressure of the weight of the body. M. Malgaigne does not altogether reject the agency of rickets in producing curvatures of the spine; he only restricts its influence to a much smaller number of cases than has generally been done. According to M. Maisonabe rickets only bear a proportion to other causes of one to three ; while alteration of the intervertebral substance, without any affection of the bones, will be met with in two out of every three cases; lastly, in 134 dissections of distorted spine, made by the same observer, two cases only were met with in which disease was confined to the ligaments of the spine, which fact clearly shows the erroneous nature of the views entertained by Dr. Harrison and others.

To show the extent to which the intervertebral substances become altered in thickness, in cases of lateral curvature, our author details an examination of a distorted spine, made by M. Cruveilhier, in which there were two opposite lateral curvatures, one in the dorsal region, having its concavity towards the left, and the other in the lumbar region, with its concavity to the right. In this case from the third dorsal vertebra to the twelfth exclusively, the bodies of the eight bones comprised in the curvature measured seventy-one lines on the concave side, and seventy-seven lines on the convex, making a difference of six lines or a twelfth. The nine fibro-cartilages measured thirteen lines and a half on the concave side of the curvature, and twenty-five lines on the convex side or nearly

* Maisonabe, Journal Clinique sur les Difformités, tom. i.

double. The inferior curvature was formed by the last dorsal and the five lumbar vertebræ. The height of these six bones on the concave side was fifty-one lines, on the convex seventy-two lines, the difference amounting to twenty-one lines, or nearly a third. The intervertebral substances included in this curvature measured twelve lines and a half on the concave side and thirty on the convex, making a difference of about three fifths.* M. Serres has published an analogous case to the above, which proves that a very great sinking of the fibro-cartilages and also of the bones takes place in cases of extensive lateral curvature of the spine. The vertebræ were here found to have diminished much more in thickness in the lumbar than the dorsal region; which Cruveilhier endeavours to explain by the support which the ribs afford to the vertebræ in the latter situation.

In the observations which he has made on this subject, our author has particularly called attention to the extent to which the intervertebral substances become altered in shape in cases of curvature of the spine; a fact which, though previously observed by other authors, had not been very particularly attended to. He has quite failed, however, in substantiating his first proposition, that this sinking of the intervertebral layers is owing to some morbid alteration of their tissue; for he describes no changes of structure whatever; and as to the idea that there has been some congenital defect of nutrition of one side of the body, in these cases, if true it is probable that the vascular and osseous systems would be affected in a greater degree than the intervertebral substances; or at least their imperfect development would have a much greater influence in causing deformity of the spine. We are inclined to conclude with Mr. John Shaw, that the alteration in shape of the intervertebral substances must be principally referred to mechanical causes.

M. Malgaigne forcibly shows the absurdity of the system of using extension in cases of curvature of the spine.

> "Suppose you apply the method of parallel extension to straighten a curvature of the spine, in which the difference between the convex and the concave sides is thirty-eight lines; if this is to be restored by elongation of the fibro-cartilages they must be stretched from thirteen lines to fifty lines, that is, four times their length. Still this is not all, for the efforts of extension act at the same time on the convex side, which will certainly be also somewhat elongated, and thus the distance will be augmented, to which the other side must be extended before the spine regain its natural form. And in short, when you have given to the fibro-cartilages fifty lines of height where they ought to possess only thirty, can it be supposed that the vertebræ will be firmly connected together by the ligaments which must also have been stretched to so great an extent?" (T. ii., p. 20.)

A curvature of the spine in one direction produces almost inevitably a second one in an opposite direction, this last being caused entirely by the action of the muscles, which act unequally for the purpose of preserving the equilibrium of the trunk. It is a point of some interest to determine, in a case where two curvatures exist, which of them is primary and which secondary. Most authors consider that distortion generally commences in the lumbar region;† but Malgaigne says that the superior is almost always the primitive curvature, the articulating sur-

* Bulletins de la Société Anatomique.

† See John Shaw on the Spine, p. 53; Lawrence, Med. Gaz., vol. vi., p. 613; Sir C. Bell, Med. Gaz., vol. v. p. 234.

faces on the bodies of the dorsal vertebræ being smaller than those in any other part of the column. The whole of the upper part of the body in this case will incline towards one side, and a secondary curvature in an inverse direction in the cervical region will not be a sufficient counterpoise: it is necessary that there should be another curvature in the lumbar region, to restore the equilibrium. But in case the primitive curvature be seated in the lumbar region, will it not be counterbalanced by a secondary dorsal curvature? M. Malgaigne says that though this may undoubtedly take place sometimes, yet in all the cases which he has investigated, the equilibrium has first been established by a secondary curvature below the first, giving rise to twisting of the pelvis and unnatural projection of the hip.

"It results from the preceding observations that lumbar curvatures may exist without distortion of the dorsal region of the spine, though such cases are very rare, in consequence of primitive deviations in the lumbar region being themselves very unfrequent. If I may be allowed to deduce any general conclusion from the facts which I have observed, I should say that primitive dorsal curvatures arise most frequently from alteration of the cartilages, and those in the lumbar region from rickets. The former principally show themselves between the ages of seven and fourteen years, and the latter in early infancy."* (Tom. ii. p. 22.)

Lateral curvatures of the spine almost exclusively arise in young persons, a circumstance which may be explained by the mode of development of the bones. At the time of birth each vertebra will be found to consist of three bony portions, a central nucleus which forms the body, and two lateral pieces which constitute the ring. The complete ossification of the vertebræ takes place at a very late period: up to about the age of eighteen years the articulating surfaces of the body are separated from the intervertebral substance by a layer of cartilage. At this period a bony layer, or epiphysis, forms immediately in contact with the intervertebral substance, but still remains distinct from the body of the bone, so that maceration separates the body of the vertebræ into three pieces. From twenty-five to thirty years of age these epiphyses become definitely united with the bone. From the age of seven to fifteen years the intervertebral substances and the cartilage to which they are united, form so large a part of the spinal column that deviations from the natural position may be produced by much slighter causes than in later years, and may also be much more easily remedied, on account of the great degree of flexibility of the spine.

*Perineum.* The surgical interest which belongs to this region has rendered it the frequent object of attention to the anatomist, and the descriptions of it have become so numerous and complicated, that it will be necessary to say a few words regarding its structnre, so as to reconcile the opinions of different authors, before we enter on the practical applications which we find in the works before us.

Malgaigne describes the following structures in the perineum proceeding from the skin towards the bladder:

* Dr. Jules Guérin, in a Memoir lately read before the Academy of Sciences on the general etiology of lateral curvatures of the spine, states it as his opinion that the greater number of these deviations arise from what he calls active muscular retraction, which is produced by some affection of the nerves or other causes, and may in some instances be cured by dividing the affected muscle. (Comptes rendus Hebdom., &c. 23 Sep., 1839; and Brit. and For. Med. Rev. No. XVI., p. 557.)

1st. The skin, to which is attached a thin layer of adipose tissue.

2d. The first layer of fascia superficialis, also connected with a considerable quantity of fat.

3d. The deep layer of fascia superficialis, separated by a very small quantity of adipose tissue from

4th. The superficial aponeurosis of the perineum.

5th. The first layer of muscles, consisting of the erector penis (ischio-cavernosus), the accelerator urinæ (bulbo-cavernosus), and the transversus perinei; which are contained between the superficial and middle aponeurosis.

6th. The middle aponeurosis, between the layers of which the transverse arteries of the perineum are contained.

7th. The deep layer of muscles, consisting of the levator ani on the sides and posterior part, and Wilson's muscles in front.

8th. The deep aponeurosis or fascia of the perineum, which is double at its front part, being continuous with what our author calls the *pubio-prostatic* ligaments, which are generally denominated the anterior ligaments of the bladder. This deep perineal fascia is only separated from the bladder by the prostate gland, and by cellular and frequently adipose tissue, which is deposited by its sides.

M. Velpeau describes six layers of fat, and fascia between the skin and muscles of the perineum, which correspond to the three layers of Malgaigne. Dupuytren, in his posthumous memoir on Lithotomy (reviewed in our Second Volume), makes five layers, three membranous and two adipose, which correspond very nearly with those mentioned by Malgaigne. He first describes a thin layer of elastic cellular tissue, as existing beneath the skin, which contains no fat in its composition, though a layer of adipose substance is found beneath it of variable thickness. This corresponds to Malgaigne's first layer of fascia superficialis, the only difference being that one author describes the adipose tissue as forming part of the layer, and the other as being distinct from it. Dupuytren next mentions a cellulo-fibrous membrane, which, he says, is continuous with the superficial fascia of the thighs and with the dartos, and which is attached to the rami of the ischia. This must be the same as Malgaigne's deep layer of fascia superficialis; a layer of fat separates this from another cellulo-fibrous membrane (Malgaigne's superficial aponeurosis), which envelopes the root of the corpus cavernosum, the bulb, and the muscles of the perineum. This membrane is also attached to the ischia, and more firmly than the preceding, of which it is only considered by Dupuytren as forming a part, the fascia splitting into two layers. Dupuytren follows the generality of anatomists in only describing a single deep fascia of the perineum, consisting, however, of two layers (Malgaigne's middle and deep aponeurosis), which arise from the margin of the pelvis and proceed towards the median line, along with the levator ani, which they inclose between them; they embrace the rectum, the neck of the bladder, and prostate, and form the boundary between the pelvis and perineum; in front they form the anterior ligaments of the bladder. This structure has been variously described by different authors, by some it has been denominated the triangular ligament, a term which can only properly be applied to the most anterior part, which is attached to the rami and inferior surface of the symphisis pubis inclosing the subpubic ligament, and through which the urethra passes. The upper layer of this membrane is continuous with the pelvic fascia, and Velpeau describes the whole deep perineal fascia as only a portion of that membrane which lines the pelvis, and he gives it the name of the recto-vesical aponeurosis. The pelvic fascia divides into two layers at the line of origin of the levator ani muscle, one of which layers is continued on the inner and the other on the outer surface of the muscle; the inner one is continued into the prostate and front of the bladder. It seems to us perfectly immaterial whether the strong cellulo-fibrous tissue, which closes the inferior aperture of the pelvis, be designated a portion of pelvic fascia or a distinct tissue, so that its attachments and situation be clearly understood.

Any one who carefully dissects the perineum will find that it is almost impossible to distinguish the different layers above referred to (especially those described as superficial to the muscles), or rather that he may make almost as many as he likes. The fact is that beneath the skin of the perineum a quantity (which varies in different subjects) of adipose tissue exists, through which a number of cellular fibres are interspersed, which are firmly attached to the rami of the ischia and pubis; this, which constitutes the different layers of superficial fascia and aponeurosis, is continuous with the dartos and the superficial fascia of the abdomen. The most external part only is continuous with the fascia superficialis of the thighs, the deeper layers being bounded by the bony margin of the inferior outlet of the pelvis at the posterior part of the perineum.

It has been thought that an acquaintance with these different layers might afford some practical information as to the course which fluids, such as urine, effused in different parts of the perineum would take: thus when the bladder is ruptured beyond the prostate, the urine will be effused into the sub-peritoneal cellular tissue, where it may extend without meeting with any obstacle into the pelvis, the iliac and lumbar regions; and the patient will inevitably sink under the extensive gangrenous inflammation which must ensue. An opening in the prostatic portion of the urethra, which extends through the fibrous envelope of the gland, may either allow the urine to escape into the same tissue as in the preceding case (should the portion of pelvic fascia reflected over the gland be ruptured), or into the sheath of the levator ani. Rupture of the membranous portion will also give rise to effusion into the sheath of the levator ani; in fact between the two portions of the deep fascia, which will dissect the rectum and also produce great and dangerous inflammation. If the rupture takes place in the bulb, or at the point of junction of the bulb and membranous portion, immediately in front of the triangular ligament, the urine should be effused beneath the superficial aponeurosis (of Malgaigne), and will find its way forwards all round the penis, but not into the scrotum. If the superficial aponeurosis gives way, or the urethra is ruptured in front of this membrane, the fluid will effuse itself into the dartos, and may extend into the groins and front of the abdomen, also backwards round the anus.

These are the anatomical deductions, but they are not completely borne out by practical observation; for the fibrous barriers which have been described are not complete, and freely allow the urine to pass from one part of the perineum to another; thus Malgaigne has remarked that when the effusion takes place beneath the superficial aponeurosis, it soon infiltrates through this membrane and invades the scrotum.

"I may here," he says, "allude to a very curious phenomenon, that it is very rare to see the urine penetrate, in the first instance, by the side of the anus, though when the patient is lying down this is the most dependent part of the perineum. M. Blandin explains this by reference to the structure and attachments of the fascia; but without absolutely denying the resistance of this aponeurosis, I must say that it is very feeble and almost cellular in many subjects; that it evidently allows the urine to transude through its anterior part into the scrotum; and lastly, I must call attention to the course which urine takes when it is effused in the subcutaneous fascia. 'Almost always,' says M. Blandin, 'we see the scrotum become sphacelated, the testicles and the penis denuded, and the skin of the anterior wall of the abdomen undermined by the urine, before a single drop of this liquid has penetrated by the side of the anus.' As after the scrotum has become infiltrated, the aponeurosis can offer no further resistance, we must look for some other cause which encourages infiltration in an anterior direction and prevents it from taking place posteriorly. This cause appears to me to be a peculiar laxity and permeability of the cellular tissue of the scrotum, and of the layers which proceed from this region towards the abdomen: while in the neighbourhood of the anus, the close connexions of the sphincter with the surrounding layers is an almost invincible obstacle to infiltration in the median line, and at the sides the adipose tissue with its closed cells does not afford a free passage for the admission of a foreign fluid. It is thus that in infiltration of the scrotum (unfortunately produced during the injection of of a hydrocele), the fluid never descends towards the anus for all its depending position; and it is also for this reason that after the lateral operation for lithotomy, any effusion which may either occur, in consequence of the wound being badly dressed, or having too narrow an external opening, scarcely ever takes place at the back, but principally in the front part of the perineum; this is especially the case if the incision has extended into the cellular tissue of the scrotum; wherefore it is very important to hold up this part so that it shall not be liable to be wounded by the knife." (Tom. ii. p. 308.)

Rupture of the bladder or urethra, leading to effusion of urine, may take place in several ways, as from external violence from a blow, excessive distention of the bladder, or from an abscess in the neighbourhood of the urethra breaking into this canal: in all these cases there will be no external outlet to the effused fluid. But extravasation may occur in consequence of an external wound penetrating the urethra or bladder, produced either accidentally or by the instrument of the surgeon. In these cases, if the wound is very narrow no effusion will generally occur; this has been observed in paracentesis of the bladder. Infiltration also is not likely to take place if the wound is large, and has a free external aperture. This latter fact should always be borne in mind in performing the different operations for lithotomy. "Provided that the external wound," says M. Malgaigne, "is large and free there is no fear of effusion. .... ....... It is therefore a good precept to give the incision in lateral operations a triangular form, so that it keeps augmenting in width from the neck of the bladder to the integuments. It is also a bad mode of dressing to apply *charpie* to the wound, or even to bring together the edges of the incision by placing the thighs in contact. The knees ought to be held apart and the wound left open." (p. 309.) In our Fourth Volume (p. 539) we have briefly noticed a paper by Mr. Chrichton, of Dundee, on the expediency of healing the wound in lithotomy by the first intention; but we do not think that the advantages which are held out by his method are sufficiently great to counterbalance the dangers to which it may give rise.

M. Malgaigne next enters into a discussion on the advantages or disadvantages of a free incision through the prostate, and the dangers of effusion of urine to which it may give rise—questions on which there is great diversity of opinion among modern surgeons. The two circumstances which are dreaded by the generality of surgeons are, on the one hand, bruising and laceration of the prostate and surrounding parts by the exertion of too much force in extraction of the stone, in consequence of the incision through the prostate and neck of the bladder being too limited; and, on the other hand, inflammation excited by the effusion of urine into the loose cellular tissue surrounding the prostate, spreading through the pelvis and to the peritoneum; which effusion has arisen from the incision having been carried completely through the prostate gland into the surrounding textures. It was principally with the view of obviating both these difficulties, and to make a large incision without transgressing the proper limits, that Baron Dupuytren instituted his new operation of cutting through both sides of the neck of the bladder and prostate with a double *lithotome caché.* Of the extent of his success in accomplishing these objects we have already stated our opinion in the review of his splendid work in our Second Volume; and to this we refer the reader: we return to the point which we have here to discuss, namely, the reality of the danger which has been apprehended by transgressing the limits of the substance of the prostate gland. The prostate, in an adult, presents the following dimensions, according to the admeasurement of M. Senn,* viz. 13 lines of height in the vertical direction, and 19 lines of width in the centre of its transverse diameter. Radii diverging from the urethra to the circumference of the gland have the following length: from the urethra directly downwards 7 to 8 lines, from the urethra directly outwards in a transverse direction about 9 lines, and from the urethra downwards and outwards from 10 to 11 lines. An incision in the last direction, which is the one followed in most of the operations for lithotomy in this country, would only admit a calculus of 33 lines in circumference. It is, however, well known that stones vastly exceeding this size have been safely extracted in numberless instances; we must therefore seek elsewhere for some explanation of this fact. In the first place, then, it must be taken into account that the prostatic tissue is capable of great dilatation without any laceration or bruising being produced. Deschamps† made a number of experiments on this power of dilatation possessed by the prostate, from which he calculated that in an adult an incision of six lines in length will suffice for the extraction of a calculus of fourteen or fifteen lines in diameter; that a stone of fifteen to twenty lines may be extracted without any *considerable laceration* through an incision of eight lines, and so on. M. Malgaigne is of opinion that these observations of Deschamps may scarcely be depended on; for, in the first place, his experiments were made on the dead subject, in whom the power of distension was much less than we have here stated, and he only supposes that the elasticity and dilatability will be much greater in the prostate in the living state from its analogy with other tissues, and also from the fact of stones having been successfully extracted of a much larger size than could ever have been drawn through any incision in the prostate of a dead person without great laceration. In the next place it must be borne in mind that "he has never once

* Referred to by Malgaigne, and probably taken from his work, Parallèle de la Taille, which we have not, however, been able to refer to.

† Traité de la Taille, tom. iii., p. 125.

verified by dissection after death the fact that there has been no laceration nor disorganization of the parts in these cases where large stones have been extracted." The larger the incision in the prostate is, the less power of dilatation will remain, according to M. Malgaigne, as the only part which can then expand under the distension is the portion of tissue which remains between the limits of the incision and the surface of the gland (the fibrous capsule being but little extensible), and this thin layer will be then very likely to give way. "I regard it," he says, "as one of the greatest dangers of the bilateral operation (Dupuytren's) that the prostate is liable to be torn at two opposite points, so that the anterior half may be completely severed from the posterior." (Tom. ii., p. 318.) When a small opening is made in the prostate the gland alone is not in danger of being bruised and lacerated, in the endeavour to extract the stone, but the bladder is also dragged forwards by the forceps, torn from its connexions, and contused about the neck.

"There is but one way, in my opinion," says Malgaigne, "to render the lateral operation for the stone less dangerous, and this is to divide the prostate freely, on one side only, entirely through, cutting the body of the bladder and the surrounding cellular tissue if the size of the calculus requires it; in fact, to make such a free aperture for the passage of the stone that the wound will remain an *incision*, and not be complicated with contusions and lacerations. Forced dilatation, however slow it may be, is wrong.......... With regard to the fears of urinary effusion, the success of the operation when performed above the pubis is sufficient to make us easy on this head. The essential point is, that the external wound be sufficiently large to open a free passage for the fluid. It seems to me to be an advantage to make an incision to a greater or less extent on the right side of the raphé, so that the cut on the left side need not extend so near to the tuberosity of the ischium. An *external bilateral* incision, and an *extensive* but *unilateral* incision through the prostate is, in a few words, the proceeding which I recommend." (Tom. ii., p. 319.)

Malgaigne's opinion is directly opposed to that of many of the most celebrated writers on lithotomy. Scarpa, Dupuytren, Brodie, Stanley, Liston, and others, think that the greatest dangers are to be apprehended from too free an incision of the prostate and neck of the bladder, infiltration of urine into the pelvis being thus liable to be occasioned. Sir B. Brodie and Mr. Stanley consider the plan of making a second incision in the right side of the prostate, in cases where the stone is very large, preferable to the extension of the cut in the left side. Mr. Liston and Mr. Key recommend, in certain cases where a large opening is required, the introduction of a bluntish gorget, such as that used by the late Mr. Martineau of Norwich, for the purpose of enlarging the incision which has been first made by the knife. The gland is thus torn through without (so Mr. Key says) being bruised, and the incision is thus enlarged as far as the deep fascia, while this membrane remains uninjured. Mr. Liston expresses himself so strongly with regard to the danger of carrying the incision through the prostate too far that we shall close the view of this side of the question in his words: "The internal incision," he says, "must be very limited indeed; it should certainly not extend beyond seven lines from the urethra outwards and downwards,......the less that is cut the greater will be the patient's safety. The object in following that method is not to interfere with the reflexion of the ileo-vesical or pelvic fascia from the sides of that cavity over the base of the gland and side of the bladder. If this natural boundary between the external and internal cellular tissue is broken-up there is scarcely a possibility of preventing infiltration of urine, which *must almost certainly prove fatal.*"*

Cheselden, Desault, John Bell, Langenbeck, and Klein (one of the most experienced operators in Germany), take the same view of the subject as M. Malgaigne; and as of the same opinion may be added Mr. Samuel Cooper, who states in his Dictionary that he does not think that the apprehension of effusion of urine, sloughing, &c., sufficiently well founded to make it advisable for surgeons to relinguish the plan of making a complete division of the side of the prostate gland, and a limited one in the neck of the bladder. When effusion does occur, he thinks that it is from the external incision being too small. He also says that Sir A. Cooper has stated to him in conversation that it is his opinion that the chances of effusion from a free incision are much less than have been apprehended.

In taking a review of these conflicting opinions it appears to us that it has not been sufficiently proved by dissection that the cause of death in unsuccessful cases is generally gangrene from effusion of urine. Mr. Key says† that in all the cases which he examined there was suppurative inflammation of the reticular texture surrounding the bladder. This we are disposed to consider as more likely to arise from force used in extracting the stone through a narrow aperture (dragging forward the prostate and neck of the bladder from their connexions) than from urine effused into the cavity of the pelvis, which would occasion gangrenous and not simply suppurative inflammation. A very free incision may be made through the prostate gland without cutting the pelvic fascia, but we do not think that this can be well accomplished by means of the lithotome caché (the instrument used by M. Malgaigne and the generality of French surgeons) or the gorget, it being impossible to vary the direction of the wound during the formation of the incision with these instruments. With the knife, however, the incision may be carried through the widest part of the prostate and even extended beyond that organ, without entering the sub-peritoneal tissue, the knife being carried first downwards and outwards and then almost directly downwards, and brought out below that part of the gland to which the pelvic fascia adheres in its passage from the levator ani to the neck of the bladder. By this mode of operating a double purpose will be gained; a free incision will be formed, and thus bruising and laceration of the prostate avoided ; while the risk (great or small) of effusion in the sub-peritoneal tissue will be avoided. After a careful investigation of this subject we are obliged to come to the conclusion that though much has been written and many opinions brought forward, little is still known of the immediate causes which occasion mischief in many unsuccessful cases ; and we would recommend surgeons carefully to examine all cases that may come under their notice, in which death occurs, chiefly with the view of ascertaining what the extent and direction of the wound has been in the prostate and neck of the bladder ; as this may throw some light on the question whether the incision has been carried too far, or whether the parts have been bruised and lacerated.

* Liston's Practical Surgery.

† On the Section of the Prostate Gland.

